# Next-generation sequencing yields the complete mitochondrial genome of the endangered Milos viper *Macrovipera schweizeri* (Reptilia, Viperidae)

**DOI:** 10.1080/23802359.2018.1532348

**Published:** 2018-10-26

**Authors:** Evanthia Thanou, Panagiotis Kornilios

**Affiliations:** a Department of Biology, University of Washington, Seattle, WA, USA;; b The Molecular Ecology Backshop, Loutraki, Greece;; c Institute of Evolutionary Biology (CSIC – Universitat Pompeu Fabra), Barcelona, Spain

**Keywords:** *Macrovipera schweizeri*, Milos viper, mitogenome, mtDNA, Squamata, Viperidae

## Abstract

The Milos viper, *Macrovipera schweizeri*, is an endangered viperid snake found on four Aegean islands (Greece). Its complete mitochondrial genome, the first reported for the genus *Macrovipera*, was assembled through next-generation sequencing. Its total length is 17,152 bp and includes 22 tRNAs, two ribosomal RNA genes, 13 protein-coding genes and two control regions, showing the typical gene-arrangement for Viperidae. Eight tRNAs and *ND3* are encoded on the light strand, while all other genes are encoded on the heavy strand. A mitogenomic phylogeny that included *Macrovipera schweizeri* and 13 other viperid genera returned an unresolved relationship among the genera *Macrovipera*, *Daboia* and *Vipera*.

*Macrovipera schweizeri* (Werner, 1935), commonly known as the Milos viper or the Cyclades blunt-nosed viper, belongs to Viperidae, a family of worldwide distribution that includes more than 300 species and 35 genera organized in three subfamilies: the true vipers Viperinae, Azemiopinae and Crotalinae (Alencar et al. 2016). The Milos viper is an endemic found only on four islands of the Milos archipelago in the west Aegean Sea (Greece) and it is one of the most iconic European reptiles mostly because of its classification as an Endangered species by the IUCN (Böhme et al. 2009). Until 2018, it was the only European snake species to be classified as Endangered, with another viper, *Vipera graeca*, being added to this category very recently. Four other cases of European snakes of important conservation status are also recognized by the IUCN, but they correspond to regionally endangered subspecies. Several complete mitogenomes have been published for viperids, with only three for true vipers. Here, we present the complete mitogenome of the Milos viper, which is also the first sequenced mitogenome of the genus *Macrovipera*.

A tissue sample (308MS) was collected from a road-killed individual on Milos Island near Pollonia (36.757370°, 24.510345°) and deposited in the Biology Department of the University of Patras (ZMUP). Genomic DNA was extracted and used, together with other squamate samples, in a comparative analysis that utilized ultraconserved elements (UCEs) under a sequence capture protocol (Faircloth et al. 2012). The 150 bp paired-end library was prepared using the Truseq Nano DNA kit (Illumina Inc., San Diego, CA) and sequenced on a single Illumina HiSeq 4000 lane.

The mitogenome was assembled de novo from the off-target sequences with NOVOPlasty 2.7.1 (Dierckxsens et al. 2017). Genes were annotated using the MITOS WebServer (Bernt et al. 2013) and checked manually. Alignment with other viperid mitogenomes, one from every genus currently available in GenBank, was done with MAFFT (Katoh et al. 2017). This dataset, after removing the two poorly aligned control regions, was used to reconstruct a Maximum Likelihood (ML) tree with IQ-TREE1.4.3 (Nguyen et al. 2015), using 1000 ultrafast bootstrap alignments (Minh et al. 2013).

The Milos viper mitogenome (Genbank accession number MH717075) has a length of 17,152 bp and includes 22 transfer RNA genes (*tRNA-Leu* and *tRNA-Ser* are duplicated), two ribosomal RNA genes, 13 protein-coding genes and two control regions, showing the typical gene-arrangement for Viperidae (IIIB type in Qian et al. 2018). The putative L-strand replication origin (*OL*) is 36 bp long and located between *tRNA-Asn* and *tRNA-Cys*. Eight tRNAs and *ND3* are encoded on the light strand, while all others are encoded on the heavy strand. The two control regions have similar length with 1020 bp (*CRI*) and 1030 bp (*CRII*). The overall composition of the heavy strand is A (32.1%), T (27.1%), C (28.3%) and G (12.5%).

The unrooted ML phylogeny based on complete mitochondrial genomes of viperid genera distinguishes the three subfamilies as monophyletic groups (Figure 1). *Macrovipera* is nested within Viperinae but it is not clear whether it is closely related to the genus *Vipera* or *Daboia*. The relationship between the latter two and *Macrovipera*+*Montivipera* have historically been unclear in molecular phylogenies (Lenk et al. 2001; Garrigues et al. 2005; Stümpel and Joger 2009; Alencar et al. 2016). It seems that even complete sequences of the mitochondrial DNA are not able to resolve these relationships which could point to a rapid radiation from the common ancestor of this group.

**Figure 1.**
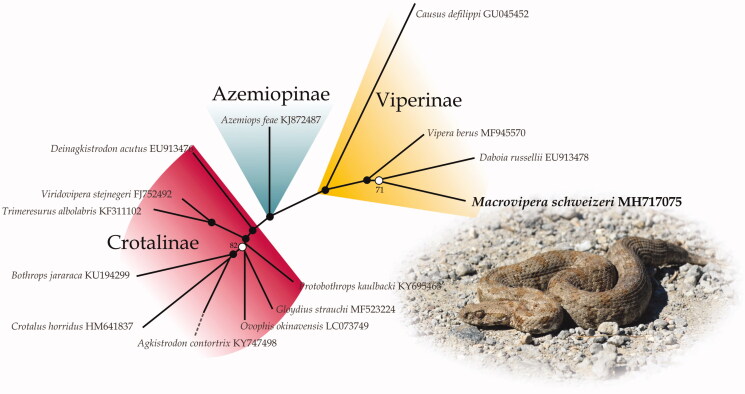
The unrooted maximum-likelihood tree based on the mitogenomes of *Macrovipera schweizeri* and 13 other viperid genera. Numbers next to nodes indicate bootstrap support values (bs): closed circles = 100 bs; no circles <70 bs; open circles >70 bs.
